# Advances in research on unphosphorylated STAT3: A review

**DOI:** 10.1097/MD.0000000000043476

**Published:** 2025-07-25

**Authors:** Lei Zhang, Wei Guo, Na Lu, Yuqing Tian, Jinghua Yang, Liang Wang

**Affiliations:** a Research Center of Traditional Chinese Medicine and Clinical Pharmacy, Shandong Provincial Maternal and Child Health Care Hospital Affiliated to Qingdao University, Jinan, China.

**Keywords:** disease, JAK-STAT, tumor, U-STAT3

## Abstract

Signal transducer and activator of transcription 3 (STAT3), a member of the STAT family, is a pivotal player in multiple cellular processes including proliferation, migration, differentiation, angiogenesis, programmed cell death, inflammatory response, and immune activation. As a transcription factor, the regulation of STAT3 activity is incredibly complicated as it is implicated signaling pathways in many distinct types of cells and under a diverse array of conditions. The activation of STAT3 commonly depends on the phosphorylation of specific residues in response to numerous growth factors and cytokines. With the discovery of STAT3 mediating gene expression in the absence of tyrosine/serine phosphorylation, a growing number of investigations related to unphosphorylated signal transducer and activator of transcription 3 (U-STAT3) demonstrated that U-STAT3 possess distinct features in regulation of gene expression. Herein, we summarize the current research progress in the roles of U-STAT3 in transcription and try to provide a basis for better understanding the complicated STAT3 activity regulation and its diverse functions.

## 1. Introduction

Signal transducer and activator of transcription (STATs) proteins are a 7-member family of cytoplasmic transcription factors (STAT1, STAT2, STAT3, STAT4, STAT5A, STAT5B, and STAT6) that transduce signals from cytokine receptors to the nucleus, where STAT dimers bind to deoxyribonucleic acid (DNA) and regulate specific gene expression.^[1,2]^ STATs were originally discovered in 1990s as DNA-binding proteins capable of mediating interferon (IFN)-dependent gene expression.^[3–5]^ STATs consist of a common N-terminal domain, a DNA-binding domain, a coiled-coil domain, a linker domain, an steroid receptor coactivator homology 2 domain (SH2) and a transactivation domain, each with distinct functions.^[6,7]^ Of the 6 domains, the SH2 domain is considered essential for dimerization and consequent nuclear translocation of STATs.^[8]^ STATs are located predominantly in the cytoplasm in the latent state until they are activated by a variety of extracellular ligands, including cytokines, growth factors, and hormones via their respective receptors.^[9]^

Among 7 STATs, STAT3 is particularly linked to inflammation and tumorigenesis.^[10]^ In 1993, STAT3 was originally discovered as an acute-phase response factor that binds to its consensus DNA motif in nuclear extracts from the liver cell line human hepatocellular carcinoma cell line 2 upon stimulation with the cytokine interleukin-6 (IL-6).^[11]^ In the following year, STAT3 protein was parallelly cloned and characterized further by 2 independent research groups and was defined as a signal transduction molecule that could be phosphorylated by IL-6 family receptors.^[12,13]^ Further studies showed that activation of STAT3 signaling can be triggered by a wide range of receptors including cytokine receptors, growth factor receptors, receptor tyrosine kinases, serine/threonine kinases, non-receptor tyrosine kinases (NRTKs), G-protein-coupled receptors, homodimeric hormone receptors, and toll-like receptors.^[14,15]^ Binding with the specific ligands, the receptor dimerizes and provides signals for Janus kinases (JAKs) or other adapter proteins to activate downstream STAT3 signaling due to conformational changes. Upon activation by phosphorylation on tyrosine 705 or serine 727 within the C-terminal transactivation domain, STAT3 separates from the receptor/kinase complex to the formation of homodimers or heterodimers through the SH2 domain.^[16]^ The phosphorylated signal transducer and activator of transcription 3 (P-STAT3) dimer then imported into the nucleus through interactions with importins thereby binding to gamma-activated sequence (GAS; interferon-γ-activated sequence) motif with a consensus palindromic DNA sequence upstream of the target gene promoter of nuclear DNA to modulate transcription of target gene.^[17]^ STAT3 signaling is supervised by negative modulators including member of protein inhibitors of activated STATs, suppressors of cytokine signaling families, and protein tyrosine phosphatases and thus implementation of negative feedback regulation.^[18]^ Additionally, other posttranslational modifications such as methylation,^[19]^ acetylation,^[20]^ ubiquitination,^[21]^ sumoylation^[22]^ and S-glutathionylation^[23]^ also participate in the regulation of STAT3 activities. CREB-binding protein/p300 acetyltransferase mediates acetylation of STAT3 at several lysine residues in SH2 and N-terminal domains, which is related to functional intensification of STAT3 in increased dimer stabilization, tyrosine phosphorylation, nuclear translocation, and localized histone hyperacetylation of target promoters.^[24]^ Similarly, methylation and sumoylation are also important in regulating the activation of STAT3. The methylation of STAT3 functions in inhibition of DNA-bound STAT3 dimers and enhancement of phosphorylation of STAT3.^[25–27]^ Sumoylation of STAT3 at the lysine 451 and 679 is strongly correlated to regulation of STAT3 phosphorylation.^[28,29]^ Though most STAT3 reviews are focused on P-STAT3, this manuscript was trying to summarize the recent research progress in the roles of unphosphorylated signal transducer and activator of transcription 3 (U-STAT3), which has equally important functions as P-STAT3 in understanding the roles of STAT3 in gene transcription as well as in the disease settings. We hope it could provide a basis for further study the complicated STAT3 activity regulation and its diverse functions.

## 2. Mechanism involving U-STAT3 in cellular regulation

### 2.1. U-STAT3 functions as a transcription factor

**Figure 1.**
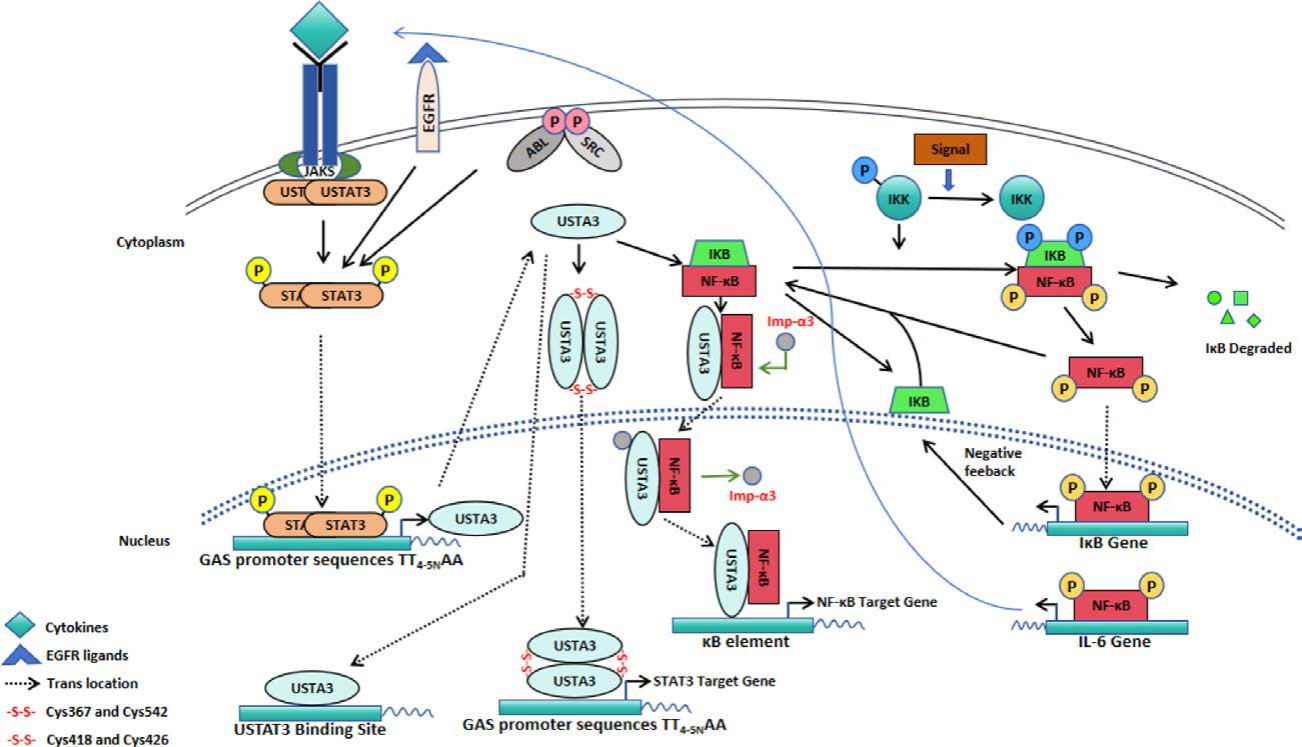
Canonical and noncanonical STAT3 signaling. Extracellular stimulation of RTKs (e.g., EGFR), non-receptor tyrosine kinases (e.g., SRC) or cytokine receptors (e.g., IL-6) can lead to canonical activation (tyrosine phosphorylation) of STAT3 for a number of cellular functions via its nuclear transcriptional function. U-STAT3, induced to a high level due to activation of the STAT3 gene in response to ligands such as IL-6, competes with IκB for NFκB p65/p50. The U-STAT3:U-NFκB complex activates promoters that have κB elements. U-STAT3 also drives the expression of some genes that do not have κB elements, by an unknown mechanism (not shown). The κB element of the IL-6 gene is driven by canonical U-NFκB signaling in response to ligands such as TNF-α or IL-1, setting up the positive feedback loop that is driven by the activation of STAT3 in response to secreted IL-6, leading to an increased level of U-STAT3 that sustains the activation of genes. ABL = Abelson, Cys = cysteine, EGFR = epidermal growth factor receptor, GAS = gamma-activated sequence, IκB = inhibitor of nuclear factor-κB, IKB = I kappa B kinase, IKK = inhibitor of kappa B kinase, Imp-α3 = importin-α3, JAKs = Janus kinases, NF-κB: nuclear factor kappa B, RTKs = receptor tyrosine kinase, SRC = steroid receptor coactivator, STAT3 = signal transducer and activator of transcription 3, U-NFκB = unphosphorylated nuclear factor kappa B, U-STAT3 = unphosphorylated signal transducer and activator of transcription 3.

Most of the research commonly focuses on canonical P-STAT3 signaling, activation of which is initiated by stimulation of receptor tyrosine kinases. The discovery of tyrosine phosphorylation-independent STAT3 mediated gene expression opens a door of exploring the significant roles of U-STAT3 in regulation of diverse biological processes.^[30]^ Like P-STAT3, U-STAT3 can also translocate into nucleus and prominently exist there at quiescent state. By reconstitution of F705-STAT3 mutant (the canonical Y705 tyrosine phosphorylation site was mutated) in STAT3 null mouse embryonic fibroblasts, Yang et al showed that U-STAT3 expression enhances the gene-inducing actions, antiviral and antiproliferative sensitivity to IFN.^[30]^ The U-STAT3 accumulates due to the strong activation of STAT3 gene by P-STAT3 dimers in response to IL-6 and other ligands that activate the gp130 common receptor subunit (Fig. 1).^[31]^ Mechanisms of U-STAT3 as a transcription factor binding to DNA was initially investigated by mutating a GAS element of the endogenous STAT3 promoter. The results demonstrate that the expression of STAT3 in response to IL-6 is dysregulated in a portion of tissues but not all, which confirmed that STAT3-dependent expression of STAT3 gene can be regulated mainly by binding to GAS element and additional elements were not recognized in this study.^[32]^ In-depth investigation on U-STAT3-DNA interactions by atomic force microscopy Imaging revealed that U-STAT3 molecules bind to the GAS DNA-binding site as dimers and monomers^[33]^ further supporting the critical role for U-STAT3 in regulation of gene expression which is consistent with previous studies.^[30,31,34]^ With the exception of GAS element, U-STAT3 binding to AT-rich DNA sequence sites was observed.^[34]^ In addition, specific DNA structures including DNA nodes and 4-way junctions can be recognized by U-STAT3 which indicates a character for U-STAT3 as a chromatin/genome organizer since these structures are crucial for chromatin organization.^[33]^ Concurrently, an 67.5-kDa U-STAT3 isoform, of which the C-terminal is truncated, was discovered for its capacity to recognize single-stranded spacers^[35]^ within cruciform structures that is also related to chromatin organization and gene expression, further supporting U-STAT3 function as a transcriptional activator and a chromatin/genomic organizer. Protein electrophoretic mobility shift assay showed that purified U-STAT3 core could bind to a high affinity STAT3 target DNA sequence and the U-STAT3:DNA interaction was then confirmed by X‐ray crystallography, and supported by circular dichroism spectroscopy and molecular dynamics simulations.^[36]^ U-STAT3 binding to a specific enhancer sequence was the first report. Although the U-STAT3 is still in the dimeric form, mutation of cysteine (Cys) 367 and Cys542 on the U-STAT3 completely abolished its DNA-binding capacity (Fig. 1), indicating that the Cys367–Cys542 disulfide bridge might induce structural changes altering the overall conformation of the dimeric species and is crucial for U-STAT3 DNA-binding activity.^[37]^ Mechanism involving U-STAT3 in cellular regulation and the relationship with the canonical STAT3 signaling pathway are summarized in Figure 1. In summary, U-STAT3 can translocate the nucleus as dimers and monomers in quiescent state and bind DNA GAS elements to regulate gene expression, in addition, the Cys367–Cys542 disulfide bridge affects the dimeric form of U-STAT3 and is essential for U-STAT3 DNA-binding activity (Fig. 1). Insight into the mechanisms by which U-STAT3 functions intracellularly such as U-STAT3 nuclear transport, U-STAT3 dimer formation, and U-STAT3 binding to DNA, will benefit researchers to target U-STAT3 for the treatment, prevention and diagnosis of clinical diseases.

### 2.2. Nuclear-cytoplasmic transport of U-STAT3

In general, active shuttling between nuclear and cytoplasmic compartments requires both efficient nuclear import and export. For U-STATs and P-STATs, the transport mechanisms may be entirely different. For most STATs, nuclear import of tyrosine-phosphorylated STAT dimers is an active process requiring energy which interact with nuclear pore proteins by carrier proteins importins α and β.^[38]^ By contrast, shuttling of U-STATs in and out of the nucleus is independent of metabolic energy, rather than by interaction with nuclear pore proteins directly.^[38]^ However, among all the STATs, only STAT3 could shuttle between the cytoplasm and nucleus independent of its phosphorylation, despite sharing domain structures and sequence of all STATs.^[39,40]^ The amino acids 150 to 162 within the coiled-coil domain of the STAT3 was proposed to be recognized by specific import carrier importin-α3 and importin-α6, with importin-α3 as the primary one because of its ubiquitous expression.^[41]^ In addition, interaction of STAT3 with importin α5 is shown to be inseparable from R214 and R215 of STAT3^[42]^ and the R214A/R215A mutant was shown to increase chromosome maintenance protein 1 recognized leucine-rich nuclear export signals-dependent nuclear export.^[43]^ However, other studies indicated that U-STAT3 nuclear shuttling was not affected by the mutation of R214 and R215.^[41,44]^ It seems likely that U-STAT3 shuttling between the cytoplasm and the nucleus is independent of binding to importin. Up to then,^[43]^ as has been reported for U-STAT1,^[45]^ whether nuclear import of U-STAT3 facilitated by interaction with constituents of the nuclear pore complex directly is still merely speculated. Afterwards, the investigation by live cell imaging studies suggest that U-STAT3 is continuously imported to the nucleus and exported independent of tyrosine phosphorylation, and nuclear import is mediated by the importin-a/importin-b1-Ran system.^[46]^ The emergence of conflicting findings on nucleocytoplasmic shuttling of U-STAT3 suggests that molecular mechanism of U-STAT3 Nuclear-cytoplasmic transport is far from clear and needed to clarify.

Among the STATs family, it is noteworthy that only STAT3 demonstrates the ability to shuttle between the cytoplasm and nucleus. Presently, the confirmed mechanism underlying the nuclear-cytoplasmic transport of U-STAT3 involves the recognition of amino acids 150 to 162 within the helix-loop-helix domain by specific import carrier proteins, notably α3, facilitating its transport. This mechanism presents a significant departure from the transport mechanism observed in phosphorylated P-STAT3, suggesting a need for further comprehensive investigation by researchers.

### 2.3. U-STAT3-dependent gene expression

Initially, STATs were taken as laten transcription factors shuttling between the cytoplasm and the nucleus to induce gene expression only in response to cytokine stimulation. As research progresses, STAT1 and STAT3 were found to be present in nucleus independence of tyrosine phosphorylation in different type of cells.^[47,48]^ Following the discovery of STAT1 in driving gene expression in the absence of tyrosine phosphorylation,^[49]^ the U-STATs as transcription factor in gene expression was extend to U-STAT3 by Yang et al^[30]^ Accumulation and increase in the level of U-STAT3 is depend on the activation of STAT3 gene expression in response to signal-dependent tyrosine phosphorylation of STAT3 due to the presence of GAS element in STAT3 promoter.^[32,50]^ P-STAT3 persists in cell for several hours, however, U-STAT3 lasts for many days after the initial activation of P-STAT3.^[30]^ It was suggested that increase in the concentration of endogenous U-STAT3 following long-term treatment with IL-6 allows U-STAT3 competes effectively with IκB for NFκB binding and forms a novel transcription factor that induces related gene expression. In an in vivo study, STAT3 and p65 were shown to physically interact and p65 homodimers can cooperate with U-STAT3 to bind to a specific type of κB motif.^[51]^ Interestingly, unphosphorylated STAT2 (U-STAT2) and IRF9 can cooperate with NF-κB to drive IL-6 expression which was recently reported by our group.^[52]^ Estrogen receptor (ER) stress induced endogenous expression of CCL5 was shown via elevating U-STAT3 expression in breast cancer cells.^[53]^ U-STAT3 modulates alpha7 nicotinic receptor (alpha7nAChR) signaling and cytokine production by cooperation with NFκB in macrophage.^[54]^ U-STAT3 is capable of mediating TGF-β1-dependent transcriptional events that leads to reorganization of the actin cytoskeleton and actin alpha 2 smooth muscle as well as monocyte chemotactic protein 1 transcription.^[55]^ However, it also reported that U-STAT3 acts as a dominant-negative inhibitor through direct interaction with p65 promoter in mesangial cells, which suppresses the ability of phosphorylated nuclear factor kappa B to induce cytokine-dependent activation of the inducible nitric oxide synthase.^[56]^ Correlated research suggested that U-STAT3 activates gene expression by a novel mechanism distinct from that used by P-STAT3 dimers. Potential genes regulated by U-STAT3 are summarized in Table 1.

**Table 1:** Potential genes regulated by U-STAT3.

Gene	Ontology
RANTES	Immune response, inflammatory response, cell proliferation, and differentiation
IL-6	Immune response and inflammatory response
IL-8	Immune response and inflammatory response
MET	Proliferation and movement and differentiation
MRAS	Cell growth, differentiation, and dysregulation
	
IFNβ	Immune response, response to pathogens, or parasites
ICAM-1	Immune response, inflammatory response, and cell metastasis
TIS11D	Cell differentiation
MAD4	Cell proliferation and differentiation
CDC2	Regulation of cell cycle and cell migration
Cyclin B1	Regulation of cell cycle
E2F-1SCYA5	Regulation of cell cycle and apoptosis
Survivin	Regulation of cell apoptosis
MNS1	Regulation of cell cycle
MPAK-3	Regulation of cell division, proliferation, migration and differentiation
TFIIH	Regulation of DNA repair
BUB1	Cell proliferation
BCL2A1	Regulation of cell apoptosis
RANGAP1	Nucleocytoplasmic transport and cell division
PLK-1	Cell division
CDC25C	Regulation of cell cycle
TACC3	Cell division, growth, differentiation, invasion, and metastasis
SOCS-2	Regulation of signal transduction
C-MYC	Regulation of apoptosis
DP1	Regulation of cell cycle
C-FOS	Cell growth, proliferation, and differentiation
C-JUN	Regulation of cell apoptosis and inflammatory response
BCL-X	Regulation of cell apoptosis
CXCL11	Immune response and inflammatory response
GADD45γ	Regulation of cell cycle and apoptosis
MYD88	Immune response and inflammatory response
T2BP	Immune response
OAS1γ	Response to virus, immune response, and regulation of ribonuclease activity
OPN	Immune response
RGS2	Regulation of the constriction and relaxation of vascular smooth muscle
CTGF	Regulation of cell division, proliferation, migration, and differentiation
ACTA1	Formation of cytoskeleton structure
α-SMA	Contraction of smooth muscle
MCP-1	Immune response and inflammatory response
MDR1	Regulation of membrane transport
NANOG	Regulation of cell proliferation and differentiation
VEGF	Regulation of vascular endothelial cell proliferation and migration

ACTA1 = actin alpha 1 skeletal muscle, BCL2A1 = B-cell lymphoma 2-related protein A1, BCL-X = B-cell lymphoma/leukemia-X, BUB1 = budding uninhibited by benzimidazoles 1, CDC2 = cell division cycle 2, CDC25C = cell division cycle 25C, C-FOS = cellular oncogene Fos, C-JUN = Jun oncogene, C-MYC = myelocytomatosis viral oncogene homolog, CTGF = connective tissue growth factor, CXCL11 = chemokine (C-X-C motif) ligand 11, DP1 = depressed palea 1, E2F-1SCYA5 = early 2 factor-1SCYA5, GADD45γ = growth arrest and DNA damage 45γ, ICAM-1 = intercellular adhesion molecule-1, IFNβ = interferon-β, IL-6 = interleukin-6, IL-8 = interleukin-8, MAD4 = mitotic arrest deficient 4, MCP-1 = monocyte chemotactic protein 1, MDR1 = multiple drug resistant gene-1, MET = mesenchymal-epithelial transition factor, MNS1 = meiosis-specific nuclear structural 1, MPAK-3 = mitogen-activated protein kinase 3, MRAS = muscle RAS oncogene homolog precursor, MYD88 = myeloid differentiation primary response gene (88), NANOG = Nanog homeobox, OAS1γ = 2′,5′-oligoadenylate synthetase 1 gamma, OPN = osteopontin, PLK-1 = Polo-like kinase 1, RANGAP1 = Ran GTPase activating protein 1, RANTES = regulated on activation normal T cell expressed and secreted, RGS2 = regulator of G-protein signaling 2, SOCS-2 = suppressor of cytokine signaling 2, T2BP = human Traf2 binding protein, TACC3 = transforming acidic coiled-coil containing protein 3, TFIIH = transcription factor IIH, TIS11D = zinc finger protein 36, U-STAT3 = unphosphorylated signal transducer and activator of transcription 3, VEGF = vascular endothelial growth factor, α-SMA = alpha-smooth muscle actin.

Among the potential genes regulated by U-STAT3, a significant proportion is associated with tumor progression. Genes including cell division cycle 2 (CDC2), cyclin B1 muscle RAS oncogene homolog precursor (MRAS), and E2F-1 are overexpressed in colon and breast carcinomas. E2F-1 is a transcription factor that activates the synthesis of messenger ribonucleic acids (mRNAs) encoding proteins needed during the cell cycle. Most E2F-1-regulated genes encode proteins involved in DNA replication or cell cycle progression.^[30]^ The products of some E2F-1-regulated genes, such as cyclin D1 and cyclin E, CDC2 and cyclin B1contribute to the G1-S and G2-M transitions and inhibit apoptosis. Recently, clear evidence has been obtained that CDC2, cyclin B1, and E2F-1 are overexpressed in mucosa-associated lymphoid tissues and head and neck carcinomas. The product of another STAT3 target gene (MRAS) contributes to cellular transformation and differentiation. The pivotal role of STAT3 in oncogene expression underscores the potential clinical significance of targeting U-STAT3 for tumor treatment. Furthermore, it suggests that there is ample untapped potential in this field for further exploration and development.

### 2.4. DNA-binding of U-STAT3 without tyrosine phosphorylation

Previous investigation on the nuclear location and dimerization of U-STAT3 revealed that tyrosine phosphorylation of STAT3 is not mandatory,^[36,57]^ which makes researchers speculate whether tyrosine phosphorylation of STAT3 is indispensable for U-STAT3 binding with DNA. STAT3 monomers and dimers were observed to interact with promoter directly at GAS motifs without tyrosine phosphorylation both in vivo and in vitro, which indicated that U-STAT3 DNA-binding activity is independent of tyrosine phosphorylation.^[33,58–60]^ The mechanisms underlying U-STAT3 binding to DNA has been revealed by atomic force microscopy imaging. U-STAT3 binds to DNA in the form of dimers and monomers, and its DNA-binding site is similar to that of P-STAT3.^[33]^ In naïve T cells, STAT3, mostly in the nonactivated, unphosphorylated (U-STAT3), can bind to forkhead box protein O (Fox) O1 or FoxO3a promoter regulating expression of FoxO1, FoxO3a, and STAT3-deficiency led to downregulation of the expression of FoxO1, FoxO3a, and FoxO-target genes (IκB and p27Kip1).^[61]^ In addition, U-STAT3 has an effect on CRE-like sites in the CCAAT/enhancer binding protein beta promoter^[62]^ and on the glucocorticoid response element,^[63]^ which lack of classical GAS motif. Less than half the genes response to accumulative U-STAT3 can also respond to TNF-α. The rest of genes response to U-STAT3 but not TNF-α probably lack functional κB elements, and 2 such genes do not require p65 in respond to U-STAT3.^[34]^ Besides, U-STAT3 also binds to AT-rich sequences which proposed as essential binding site for c-Jun and STAT3 gene activation within the α2-macroglobulin promoter in supercoiled DNA.^[33,64]^ U-STAT3 is reported to be capable of competing with IκB protein and binding to unphosphorylated NFκB since plenty of U-STAT3-responsive gene have κB elements. The U-STAT3/p65/p50 complex accumulates in the nucleus with the aid of nuclear localization signal of STAT3, activating a subset of κB-dependent genes.^[34]^ In addition, cooperative interactions between U-STAT3 and U-NF-kB p65 was confirmed to promote heterodimer binding to GAS motif.^[51]^ Besides NFκB, U-STAT3 may interact with one or more transcription factors productively to drive the expression of this class of genes. Earlier research found that U-STAT3 combined with CRE-binding protein binds on the JunB promoter^[50]^ and combined with c-Jun on the α2-macrogloblin acute-phase response factor element.^[65]^ Other investigations show that Jun activation domain-binding protein 1 (JAB1) interacted with U-STAT3 as a direct binding partner in human colon cancer cell line COLO205, and JAB1 positively regulates U-STAT3 DNA-binding activity through protein–protein interaction. It is worth noting that the interaction of JAB1 and U-STAT3 significantly elevated the transcription level of STAT3 target genes multiple drug resistant gene-1, NANOG, and vascular endothelial growth factor.^[60]^ These findings suggest that the interaction of JAB1 with U-STAT3 but not P-STAT3 may play critical roles in oncogenesis. Furthermore, relationship between JAB1 and STAT3 related to tumorigenesis deserve further exploration and may reinforce the understanding of their regulatory mechanism by U-STAT3 and JAB1 which result in the development of effective cancer therapy. Moreover, further research can implement on recognition and elucidation of these factors interaction with U-STAT3, from which unexpected and pioneering results may arise.

Collectively, canonical activation of STAT3, involving STAT3 tyrosine phosphorylation, homodimerization, and translocation into the nucleus, in addition to a variety of target genes, also signals transcription of the STAT3 promoter, generating a pool of U-STAT3 in addition to P-STAT3. Noncanonical U-STAT3 signaling involves the activation of downstream STAT3 target genes through monomer and dimer binding to DNA.^[30,31,36]^ U-STAT3 homodimerization or heterodimerization with U-NF-kB permits phosphorylation-independent nuclear translocation of the complex.^[31,50]^ Upon nuclear translocation, homodimerized U-STAT3 complexes can bind to GAS promoter sequences (TT4-5NAA) to promote transcription of STAT3 target genes,^[33]^ whereas U-STAT3/U-NF-kB heterodimers bind to canonical NF-kB DNA-binding sites to promote transcription of NF-kB target genes.^[31,66]^ Basically, noncanonical STAT3 signaling pathways have oncogenic functions to promote cell survival and tumorigenic capabilities in a number of cancer cell types.

## 3. Exploring the multifaceted role of U-STAT3 in disease pathogenesis

### 3.1. Insights into U-STAT3 involvement in cardiac pathology

In angiotensin II type 1 receptor-induced (AT1R) cardiac hypertrophy, U-STAT3 accumulates in nucleus in response to unregulated STAT3 expression due to chronic activation of AT1R. Afterwards, U-STAT3 associates with transcription coactivator p300/CREB-binding protein and binds directly to a specific sequence site on osteopontin (OPN) promoter, which in turn, facilitates then transcription of OPN.^[67]^ Apart from OPN, other U-STAT3 potential target genes, including actin alpha 1 skeletal muscle, connective tissue growth factor, and regulator of G-protein signaling 2, are shown to be significantly correlated with progression of cardiac hypertrophy thus affecting pathogenesis of heart failure.^[67]^ Therefore, it was hypothesized that U-STAT3 is closely related to the degree of hypertrophy since U-STAT3 triggers a subset of inflammatory and pro-hypertrophic genes such as OPN, connective tissue growth factor and transforming growth factor beta in the heart.^[67]^ Despite the vital roles of U-STAT3 in cardiac hypertrophy, the effect of P-STAT3 can not be ignored since the function of P-STAT3 may be inhibited by overexpression of U-STAT3.^[51]^ The gene regulatory mechanism mediated by U-STAT3 must interplay with a broad of signaling pathway related to cardiac remodeling^[68]^ and the accumulation of STAT3 might be a potential prognostic marker for the disease. Whereas, whether accumulation of U-STAT3 can be observed in other types of cardiac hypertrophy differ from AT1R-induced cardiac hypertrophy acquires further exploration.

### 3.2. Insights into U-STAT3 involvement in sepsis

In sepsis, alpha7nAChR signaling controls the production of tumor necrosis factor (TNF) in macrophage which is believed to be a major factor triggering septic shock^[69]^ U-STAT3 but not P-STAT3 contributes to the reduction of TNF production and modulates alpha7nAChR signaling. Conversely, the production of cytokine TNF is significantly elevated and the alpha7nAChR signaling is abrogated in the absence of U-STAT3.^[54]^ The discovery that U-STAT3 regulates cytokine TNF production and alpha7nAChR signaling highlights the importance of U-STAT3 in increasing survival, aiding to cholinergic anti-inflammation and preventing systemic inflammation in sepsis.^[54]^ Meanwhile, the findings suggest that U-STAT3 and NF-kB pathway interact synergistically to regulate the innate immune responses to bacterial infection which is consistent with the presence of molecular crosstalk between STAT3 and NF-kB pathways.^[31,70,71]^ Understanding the novel and valuable aspects of U-STAT3 in immune-related implications could lead to new therapeutic approaches and more expansive research field.

### 3.3. Insights into U-STAT3 involvement in acute renal ischemic

Long-term exposure to nicotine (NIC) aggravates acute kidney injury (AKI) that could promote chronic kidney disease development and deterioration.^[72]^ U-STAT3 is also shown to be enhanced in ischemia-reperfusion-induced acute kidney injury.^[73]^ The accumulation of IR-induced U-STAT3 is significantly elevated due to chronic NIC exposure and participates in mediation of TGFβ1-dependent genes transcription resulting in accumulation of actin alpha 2 smooth muscle, fibronectin, vimentin and monocyte chemotactic protein 1, thus contributes to inflammation and actin cytoskeleton reorganization in fibrosis.^[55]^ U-STAT3 was observed to accumulate in response to IL-6,^[31]^ under insulin receptor conditions, this accumulation was further augmented in kidney.^[55]^ Similar to nicotine response in macrophage,^[54]^ U-STAT3 accumulates in response to chronic nicotine exposure on renal injury is also alpha7-nAChR dependent in porcine renal proximal tubule cell line.^[55]^ Under smoking/chronic NIC exposure, U-STAT3 expression also showed enhancement in cultured oral keratinocytes.^[74]^ Despite the different biological effects of U-STAT3 in various cell types, a strong correlation was observed between U-STAT3 and alpha7-nAChR signaling.

### 3.4. Insights into U-STAT3 involvement in subarachnoid hemorrhage

Subarachnoid hemorrhage (SAH), an acute fatal disease caused by the malformation of vascular and the rupture of arterial, can induce delayed cerebral ischemia associated with pathologic vasoconstriction and exacerbated neurologic damage.^[75]^ The identification of possible regulatory molecules in SAH pathophysiology was first deduced by gene network analysis.^[14]^ U-STAT3 was identified as the most significantly enriched regulatory factors related to SAH and its crucial role in regulation the gene expression that are involved in the late cerebral ischemia and related pathogenesis after SAH were studied in detail.^[76]^ Disruption the U-STAT3-mediated gene transcription could be a promising therapeutic strategies for SAH.

### 3.5. Insights into U-STAT3 involvement in antiviral immune responses

Type I interferon (I-IFN) plays an essential role in antiviral immune responses through the induction of interferon-stimulated genes via the JAK-STAT signaling pathway. Despite functional importance of STAT1 and STAT2, U-STAT3 is equally indispensable in the I-IFN response pathway. U-STAT3 regulates the antiproliferative, antiviral, and gene-inducing actions of I-IFN in different types of cells such as B-lymphoblastoid cells and fibroblasts.^[77]^ By binding with GAS element, U-STAT3 regulates the induction of stimulated genes (ISGs) such as CXCL11, growth arrest and DNA damage 45γ, human Traf2 binding protein, Oas1γ and myeloid differentiation primary response gene (88), by U-STAT3 homodimers as well as heterodimers with U-STAT1. U-STAT3 is shown to bind to select ISG promoters with lower affinity compared to P-STAT3. The binding of U-STAT3 may involve an indirect interaction mediated by the binding of other transcription factors since STAT1 was also recruited to the promoter regions of some STAT3-regulated genes.^[77]^ In addition, the recruitment of U-STAT3 to ISG promoters may be influenced by other transcription factors binding to sites that are adjacent to GAS elements, such as NFκB, ISRE, and IRF binding sites, since NF-κB binding site are frequently found near ISRE or GAS elements in ISG promoters.^[78,79]^

Overall, U-STAT3 plays a significant role in numerous non-tumor diseases, including cardiovascular and cerebrovascular diseases, kidney disease, and viral infections. Its importance extends to the realms of diagnosis, treatment, prognosis, and drug development for these conditions, warranting thorough investigation and discussion.

## 4. Exploring the tumorigenic potential of U-STAT3

The high level of U-STAT3 persists for a long time due to the exposure of cells to IL-6 or other pro-oncogenic stimuli.^[31]^ Increasing evidence suggests that U-STAT3 being a transcription factor plays an essential role in oncogenesis.^[31,53,80–82]^ The constitutive activation of STAT3 mediating the expression tumor-associated genes is common in a variety of tumors which enables the IL-6 derived STAT3 feedback signaling cascade.^[83]^ The accumulation of U-STAT3 accompanied with transcriptional activation of the STAT3 which is considered as modulator in oncogenic signaling in tumor that in turn exhibit constitutively activated STAT3.^[34]^ In response to IL-6, the induced U-STAT3 consequently enhanced the second waves of gene expression including regulated on activation normal T cell expressed and secreted, IL-6, interleukin-8, mesenchymal-epithelial transition factor (MET), and MARS at the transcriptional levels.^[30,31]^ The high gene expression (such as MRAS, MET, cyclin B1, and E2f1) in response to high levels of U-STAT3 but not P-STAT3 homodimers was found in diverse cancer types including head and neck squamous cell carcinomas and breast cancers.^[27]^ U-STAT3 can enhance the RAS-dependent malignant transformation in human breast epithelial cells and the growth of RAS-transformed tumors in mice was abrogated without STAT3.^[84]^ Expression of U-STAT3 enhances hepatocellular carcinoma formation in RAS-transformed hepatocytes.^[31,82]^

Up-regulation of C-C motif chemokine ligand 5 (CCL5) is a critical signature in the genesis and progression of breast cancer. The augment of CCL5, STAT3 and ER protein C/EBP homologous protein expression was observed in in breast cancer tissues compared with the normal breast epithelial tissues.^[53]^ Under ER stress, CCL5 expression was positively related to U-STAT3 levels but negatively related to that of P-STAT3. Moreover, in breast cancer cell, the accumulation of U-STAT3 in response to ER stress induces endogenous expression of CCL5 and inhibition of CCL5 secretion leds to decrease in transmigration of breast cancer cells.^[53]^ IL-6-induced U-STAT3 accumulation also leads to an increase in CCL5 expression.^[30,31]^ It is worth emphasizing that the accumulation of U-STAT3 seems contribute to inhibit the transmigration of breast cancer cells which reminds researchers the beneficial side of the U-STAT3 should be taken into account.

U-STAT3 expression is pronounced and likely plays a significant role in certain types of malignancy. In a study of 303 non-small cell lung carcinomas (NSCLC) patient samples, 91.44% of NSCLC expressed U-STAT3, whereas 60.53% of them expressed P-STAT3.^[80]^ In the study of 44 malignant mesotheliomas (MM), the expression of U-STAT3 was also higher than P-STAT3 (U-STAT3 79.5% and P-STAT3 61.4%, respectively).^[80]^ Intensive research in NSCLC and MM showed that U-STAT3 directly disrupts the interaction between microtubules and Stathmin (a small tubulin-binding protein).^[80]^ Thus U-STAT3 promotes cell migration in NSCLC and MM since Stathmin contributes to cell motility as a microtubule depolarization factor.^[85–87]^ Whereas, recent studies indicate that U-STAT3 promotes heterochromatin formation by associating with heterochromatin protein 1 and suppresses tumor progression in NSCLC,^[88]^ which consists with reports that STAT3 protein as a tumor suppressor and STAT3 deletion enhances lung cancer cell growth.^[89]^ Therefore, like STAT3, U-STAT3 also plays dual functions in tumors, at least in lung cancer, high expression of U-STAT3 contributes to tumor motility, invasion and metastasis, meanwhile, U-STAT3 also could promote heterochromatin formation and inhibit tumor growth.

The role of STAT3 in cancer has been assumed until now to be due commonly to the activation of gene expression in response to STAT3 dimers, formed through tyrosine phosphorylation in response to oncogenic tyrosine kinases such as steroid receptor coactivator. It has become evident that the constitutive activation of STAT3 drives in turn the constitutive overexpression of STAT3, and it is possible that stimuli other than STAT3 dimers can also drive STAT3 expression. To analyze the impact of gene expression driven by U-STAT3 in tumors, it would be beneficial to determine the relative expression levels of proteins driven by the total amount of STAT3 versus STAT3 dimers. This approach can aid in distinguishing between different mechanisms across various tumor types. For instance, if genes responsive to U-STAT3 are expressed in certain cancers while those responsive to STAT3 dimers are not, it would suggest that STAT3 overexpression in these cases is triggered by a signal distinct from STAT3 dimers. Such insights would be instrumental in guiding the development of drugs targeting the formation or function of STAT3 dimers.

## 5. Potential clinical implications of targeting U-STAT3 in therapeutic strategies

The U-STAT3 to support the expression of many genes in response to angiotensin II depends upon the acetylation of Lys685,^[90]^ consistent with a previous study^[67,91]^ that activation of the AT1R leads to nuclear accumulation of U-STAT3 and promotes its interaction with the acetyl transferase p300, inducing the expression of proteins involved in cardiac hypertrophy and dysfunction. Furthermore, Samraj et al^[76]^ have found that the accumulation of U-STAT3 in cerebral arteries plays a pivotal role in orchestrating the expression of genes that are involved in late cerebral ischemia and related pathogenesis following a SAH. These studies provide evidence that U-STAT3 is involved in important pathological situations outside of cancer, and from the clinical perspective, it can be considered as one of the indicators for the early diagnosis of cardiovascular and cerebrovascular diseases and to judge the effect of prevention and treatment. These provide a potential clinical possibilities for targeting the acetylation of Lys685 of U-STAT3 and inhibition of U-STAT3 binding to p300 for cardiovascular and cerebrovascular diseases therapy.

In non-small cell lung cancer, hyaluronan promotes the CD44-dependent association of the stem cell marker NANOG with U-STAT3, resulting in chemo-resistance that depends upon induced expression of the microRNA miR-21,^[66]^ which indicated the NANOG/U-STAT3 signaling specific mechanism involved in miR-21 production is significant for the formation of future intervention strategies in the treatment of CD44-activated head and neck squamous cell carcinoma and reversing drug resistance during chemotherapy. Clinically, U-STAT 3 appears to have a relationship with viral disease. Research suggested that treatment of a Burkitt lymphoma-derived Epstein-Barr virus positive cell line with a lytic cycle-inducing agent results in heterogeneous changes in cellular gene expression in refractory and lytic cells^[92]^ and provided evidence for U-STAT3 as 1 marker of the cells refractory to EBV lytic cycle induction. In addition, U-STAT3 expression enhances the sensitivity of mouse embryonic fibroblasts to the antiviral and antiproliferative actions of IFN,^[77]^ which provides a theoretical basis for the development of antiviral drugs targeting U-STAT3 in clinical treatment. In tumors, the potential clinical significance of U-STAT3 is multifaceted, the transcription of mRNAs signaled from the overexpression of U-STAT3, including cyclin B1, E2f1, MRAS, and MET, were reported to be highly expressed in numerous cancer types including breast cancers and head and neck squamous cell carcinomas.^[30]^ It indicated that U-STAT3 is expected to have diagnostic significance in the early detection and tumor progression in breast cancers and head and neck squamous cell carcinomas, while promising to be a clinically effective target for the treatment of in breast cancers and head and neck squamous cell carcinomas. In other investigations, Jun activation domain-binding protein 1 (JAB1) was identified as a direct binding partner of U-STAT3 in the nucleus of the human colon cancer.^[60]^ Clinical researchers can focus on intervening on the combination between JAB 1 and U-STAT3 to control the course of colon cancer. In human glioblastoma, the correlation of U-STAT3 with worse prognosis suggests that U-STAT 3 can be used clinically used as a prognostic marker for glioblastoma treatment.^[58]^ Most investigations have shown a positive correlation between high expression of U-STAT3 and tumor progression, whereas in lung cancer, U-STAT3 has a function in promoting heterochromatin formation, suppressing cell proliferation in vitro, and suppressing tumor growth in vivo,^[88,89]^ it suggests the multiple functions of U-STAT 3 and the complexity in tumor regulation. Clinically, different prevention and diagnosis and treatment strategies are adopted for U-STAT 3 in different tumor types.

Targeting U-STAT3 in therapeutic strategies presents a promising approach with broad clinical implications across multiple medical disciplines. By focusing on this specific form of STAT3, therapeutic interventions can potentially modulate crucial cellular processes involved in disease progression and treatment response. Further research and clinical trials are warranted to fully elucidate the therapeutic potential of this strategy and its impact on patient outcomes. Moreover, The development of targeted therapies and diagnostic tools based on U-STAT3 could pave the way for more personalized and effective cancer treatments. However, further clinical researches are necessary to fully understand the mechanisms and validate the efficacy of such approaches.

## 6. Interaction pattern between U-STAT3 and other molecules

STAT3 exerts its influence on Pol III activity by linking with miR-106a-5p to regulate transcription.^[93]^ This interaction is crucial for activating Pol III-dependent transcription, which is essential for the synthesis of certain types of RNA, including tRNAs, 5S rRNA, and small RNAs. The activation of Pol III-directed transcription by STAT3 through miR-106a-5p is a key mechanism in regulating cellular processes. TP73 is a tumor protein p73 isoform that is involved in various cellular functions, including cell cycle regulation, apoptosis, and DNA repair. It has been shown that TP73 can be targeted by miR-106a-5p, leading to a reduction in TP73 expression.^[93]^ This reduction in TP73 expression, mediated by miR-106a-5p, is significant as it impacts the balance of proteins that regulate cell growth and death, potentially contributing to cancer cell proliferative activity. Additionally, STAT3 can indirectly modulate Pol III activity through the regulation of upstream signaling pathways and transcription factors.^[94]^ For example, STAT3 activation by cytokine signaling pathways can lead to the induction of downstream effectors, including c-Myc and NF-κB, which in turn promote the transcription of Pol III-transcribed genes.^[95]^ Activation of STAT3 also signals transcription of the STAT3 promoter, generating a pool of U-STAT3 in addition to P-STAT3. Noncanonical U-STAT3-mediated signaling also activates downstream STAT3 target genes and promotes the transcription of Pol III-transcribed genes.^[30,31]^ This indirect regulation amplifies the impact of STAT3 on Pol III activity and further underscores its importance in cellular physiology and pathology.

The interaction between miR-106a-5p and TP73 is characterized by miR-106a-5p binding to the 3′ untranslated region (3′ UTR) of the TP73 mRNA. This binding leads to the degradation of the TP73 mRNA or the inhibition of its translation, thereby reducing the levels of TP73 protein in the cell.^[93]^ This regulatory mechanism is highly dynamic and complex, involving specific recognition of double-stranded DNA by transcription initiation factors, which then recruit Pol III to the transcription start site.^[96]^

The interplay between STAT3, miR-106a-5p, and TP73 is of significant importance in the field of molecular medicine. Understanding these interactions can provide insights into the molecular mechanisms underlying various diseases, particularly cancer. The ability to modulate the expression of TP73 through miR-106a-5p offers potential therapeutic targets for the development of treatments aimed at controlling cell proliferation and other related pathologies.^[93]^

In summary, STAT3 influences Pol III activity through its interaction with miR-106a-5p, which in turn targets TP73 mRNA, leading to reduced TP73 expression. This intricate regulatory network is vital for maintaining cellular homeostasis and has implications for the development of molecular medicine strategies to combat diseases such as cancer. In the field of molecular medicine, the miR-106a-5p/TP73 axis has garnered significant interest due to its implications in cancer biology and therapeutic potential. Dysregulation of this axis has been implicated in various cancers, where aberrant expression of miR-106a-5p or TP73 contributes to tumor development, progression, and therapy resistance. Targeting this regulatory axis holds promise for the development of novel diagnostic biomarkers and therapeutic strategies for cancer treatment. Overall, understanding the intricate crosstalk between STAT3, Pol III activity, TP73, and miR-106a-5p provides valuable insights into the molecular mechanisms underlying cancer pathogenesis and unveils potential targets for precision medicine interventions.

## 7. Conclusions and perspectives

Exploring the therapeutic potential of targeting U-STAT3 presents a promising avenue for novel cancer and immunomodulatory interventions, although current research in this area remains relatively nascent. By elucidating the distinct transcriptional regulatory mechanisms employed by U-STAT3, researchers can identify unique vulnerabilities that could be exploited for therapeutic benefit.

In the context of cancer therapy, traditional approaches have primarily focused on inhibiting P-STAT3 dimers to disrupt oncogenic signaling pathways. However, targeting U-STAT3 offers an alternative strategy that could complement existing therapies or overcome resistance mechanisms. Small molecules or biologics designed to selectively inhibit the transcriptional activity of U-STAT3 could potentially impede the expression of pro-tumorigenic genes, thereby inhibiting tumor growth and metastasis. Moreover, since U-STAT3 is often constitutively activated in cancer cells, targeting this unphosphorylated form may offer a more specific and durable therapeutic response compared to targeting P-STAT3 alone.

In the realm of immunotherapy, manipulating U-STAT3 activity holds promise for modulating immune responses and enhancing therapeutic outcomes. By selectively targeting U-STAT3 in immune cells, such as T cells, dendritic cells, or myeloid-derived suppressor cells, it may be possible to fine-tune immune activation and suppress immunosuppressive mechanisms within the tumor microenvironment. This approach could potentially enhance the efficacy of immune checkpoint inhibitors, adoptive cell therapies, or cancer vaccines by overcoming immune evasion strategies employed by tumors.

However, it is important to acknowledge that the development of U-STAT3-targeted therapeutics faces several challenges, including the need for precise and selective inhibitors that do not interfere with the physiological functions of P-STAT3 or other members of the STAT family. Additionally, further research is needed to elucidate the specific downstream target genes and pathways regulated by U-STAT3 in different disease contexts, as well as to validate the therapeutic efficacy of U-STAT3 inhibition in preclinical models and clinical trials. While targeting U-STAT3 represents a promising strategy for cancer and immunotherapy, further investigation is warranted to fully understand its clinical implications and therapeutic potential. Collaborative efforts between researchers, clinicians, and pharmaceutical companies will be essential for advancing this exciting area of translational research and ultimately improving patient outcomes.

Despite the significant and sustained upward trend of study on U-STAT3 in recent years, the functions of U-STAT3 in diverse biological processes still remain largely unknown. In cell culture systems, the accumulation of U-STAT3 is increased in response to IL-6 exposure, whereas the ability of IL-6 to increase U-STAT3 accumulation was abrogated not in all tissues by mutating GAS element of endogenous STAT3 promoter. It seems that additional elements differing from GAS elements maybe a breakthrough in U-STAT3-dependent expression of the STAT3 gene. Moreover, it is still not clear whether other cytokines that activate STAT3 through the common gp130 receptor subunit can lead to accumulation of U-STAT3, and whether sustained activation of STAT3 promote U-STAT3 accumulation by other means will cause a similar indirect response since STAT3 is overexpressed in many tumor cells. Whether there is a balance between P-STAT3 and U-STAT3 during cellular homeostasis and disruption of the homeostasis causing oncogenesis deserves further investigation. Although recent studies have shed light on the U-STAT3 with potential functional activities, further researches are needed to fully elucidate the physiological and pathological functions of U-STAT3.

## Author contributions

**Investigation:** Na Lu, Yuqing Tian.

**Methodology:** Jinghua Yang.

**Supervision:** Wei Guo, Liang Wang.

**Writing – original draft:** Lei Zhang.

**Writing – review & editing:** Wei Guo, Liang Wang.
